# Template-controlled piezoactivity of ZnO thin films grown via a bioinspired approach

**DOI:** 10.3762/bjnano.8.32

**Published:** 2017-01-30

**Authors:** Nina J Blumenstein, Fabian Streb, Stefan Walheim, Thomas Schimmel, Zaklina Burghard, Joachim Bill

**Affiliations:** 1 Institute for Materials Science, University of Stuttgart, Heisenbergstraße 3, Stuttgart, D-70569, Germany; 2 Institute of Nanotechnology, Karlsruhe Institute of Technology (KIT), Hermann-von-Helmholtz-Platz 1, Eggenstein-Leopoldshafen, D-76344, Germany; 3 Institute of Applied Physics and Center for Functional Nanostructures, Karlsruhe Institute of Technology (KIT), Wolfgang-Gaede-Strasse 1, Karlsruhe, D-76131, Germany

**Keywords:** piezoresponse force microscopy, template-controlled deposition, ZnO

## Abstract

Biomaterials are used as model systems for the deposition of functional inorganic materials under mild reaction conditions where organic templates direct the deposition process. In this study, this principle was adapted for the formation of piezoelectric ZnO thin films. The influence of two different organic templates (namely, a carboxylate-terminated self-assembled monolayer and a sulfonate-terminated polyelectrolyte multilayer) on the deposition and therefore on the piezoelectric performance was investigated. While the low negative charge of the COOH-SAM is not able to support oriented attachment of the particles, the strongly negatively charged sulfonated polyelectrolyte leads to texturing of the ZnO film. This texture enables a piezoelectric performance of the material which was measured by piezoresponse force microscopy. This study shows that it is possible to tune the piezoelectric properties of ZnO by applying templates with different functionalities.

## Introduction

Zinc oxide is a wide band gap semiconductor. Thin films of it can be applied in, e.g., LEDs [1–3] or transistors [4–6]. Furthermore, due to its piezoelectricity, it can be incorporated in actuators [7] or, more recently, energy harvesting devices [8–12]. ZnO crystallizes in the wurtzite hexagonal crystal structure. Its [001] and [00−1] faces are polar, since they are terminated with Zn^2+^ or O^2−^ ions, respectively. The presence of these polar lattice planes in addition to the non-centrosymmetric lattice leads to an intrinsic dipole moment that causes the piezoelectric properties of the material [7]. The electromechanical coupling can be described by a piezoelectric tensor with three independent components, namely *d*_33_, *d*_13_ and *d*_15_. For ZnO, the first one is the highest and is defined as the coupling of the materials response in the *z*-direction to an electric field applied in the same direction [7]. For piezoactive, polycrystalline ZnO thin films it is therefore essential that most of the crystallites are oriented in the same way. This means a (002) texture is formed resulting in a high mechanical deformation [13].

Growth of such oriented films was achieved via technically sophisticated methods under harsh reaction conditions [14–17]. For example radio-frequency magnetron sputtering [14,17], pulsed laser deposition [16] or sol–gel methods followed by annealing [15] were applied. Another approach is bioinspired mineralization. Here, principles from nature are adapted to deposit inorganic materials under mild reaction conditions. The crystal growth is controlled by organic additives in the mineralization solution that act as structure directing agents [18–22]. Additionally, organic templates are used to modify the surface of the substrate. Especially self-assembled monolayers (SAMs) [23–24], thin polymer brushes [25] or polyelectrolyte multilayers (PEL) [26] were successfully applied for the deposition of ZnO thin films. With these molecules, a multitude of different functionalities are available, e.g., amino-, carboxylate or sulfonate groups for hydrophilic or alkyl groups and fluorine atoms for hydrophobic modifications. The properties of these functional groups control the interaction with ZnO. In the case of a template with polar functionality, electrostatic attraction promotes attachment of the ZnO particles. On the other hand, non-polar molecules inhibit adsorption and film growth. In this way the growth and morphology of the films can be easily controlled [27–29]. Moreover, it is possible to achieve site-selective growth of ZnO by using two templates with different functionalities [25,29–35] or to change the roughness of the growing films [36].

The principle of these methods was adapted from nature where multifunctional materials are produced under ambient conditions. Mollusks for example produce the organic/inorganic composite nacre with its remarkable mechanical stability [37]. The growth of the inorganic, polycrystalline aragonite platelets is directed by biopolymers. This organic template leads to oriented attachment of the CaCO_3_ crystallites so that a preferred orientation along the *c*-axis arises [38]. According to this model system, we deposit ZnO via bioinspired mineralization onto templates with different functionalities. In our recent work [39] we have demonstrated that the piezoactivity of the grown ZnO film strongly depends on the template. In particular, the impact of a piezoactive template (i.e., a layer of tobacco mosaic viruses) on the mineralization processes of ZnO films has been investigated and an extraordinary high degree of orientation was observed. In this study, we elucidate the influence of the negative charge density of two non-piezoelectric templates on the deposition of ZnO films from water-free reaction solution. The first template is a carboxy-terminated SAM, the second consists of a PEL with polystyrene sulfonate (PSS) as a top layer. The grown ZnO thin films are characterized with piezoresponse force microscopy (PFM) to investigate the influence of the templates on the piezoelectric performance.

## Results and Discussion

**Figure 1:**
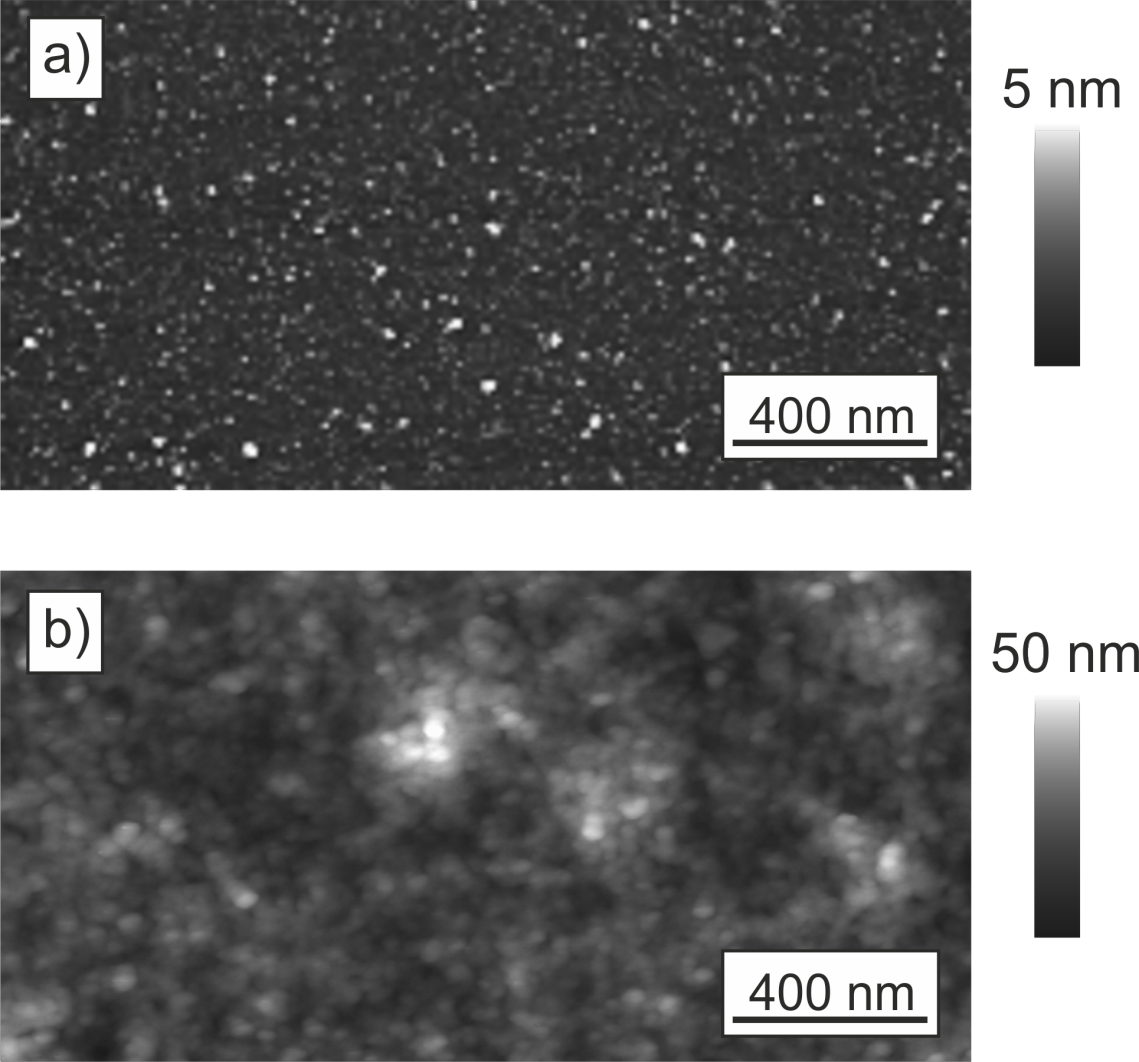
AFM height images of the COOH-SAM (a) and the PEL (b).

ZnO thin films were deposited from solution onto COOH-SAM and sulfonate-terminated PEL. The templates direct the film growth and influence the properties of the ZnO. For a better understanding of the interaction between the ZnO particles and the templates, we characterized the structure and hydrophilic character of the templates by atomic force microscopy (AFM) and water contact angle measurements (WCA) prior to mineralization. The AFM images of the PEL and COOH-SAM before mineralization show a smooth surface for both templates (Figure 1). The PEL is slightly rougher (root mean squared (rms) roughness: 6.3 nm) than the COOH-SAM (rms roughness: 0.7 nm). The SAM consists of a single, highly ordered molecular layer, whereas the PEL is composed of several layers of polyions (refer to experimental part for detailed information). The structure of the multilayer depends on the conformation of the polyion molecules and the roughness increases with every polyion layer.

WCA measurements on the PEL give a value of 29°, which is lower than the WCA of the COOH-SAM (35°). This can be attributed to a difference in polarity of the two templates. Shyue et al. measured the zeta potential of SAMs with COOH- and SO_3_H-functional groups in dependence on the pH [40]. They found that the sulfonate SAM is more negatively charged (−115 mV) compared to the carboxylate one (−75 mV) at a pH of 9, which corresponds to the pH of the mineralization solution used in this study. This indicates that the sulfonate functionality leads to a highly polar surface of the template, whereas the carboxylate is less negatively charged. However, it has to be taken into account that the deposition experiments take place in methanol. The solvent influences the effective surface charge of the substrate and particles in the solution and therefore, the interaction between both is affected. For example it was found that silica shows a decreasing surface potential if methanol is added to an aqueous electrolyte solution [41–42]. This can be explained by the lower ability of methanol to stabilize ions as can be seen from the p*K*_a_ values reported by Rived et al. [43]. Therefore, it is probable that the surface charge of the used templates is lower in methanol than it could be expected from zeta potential measurements in water.

**Figure 2:**
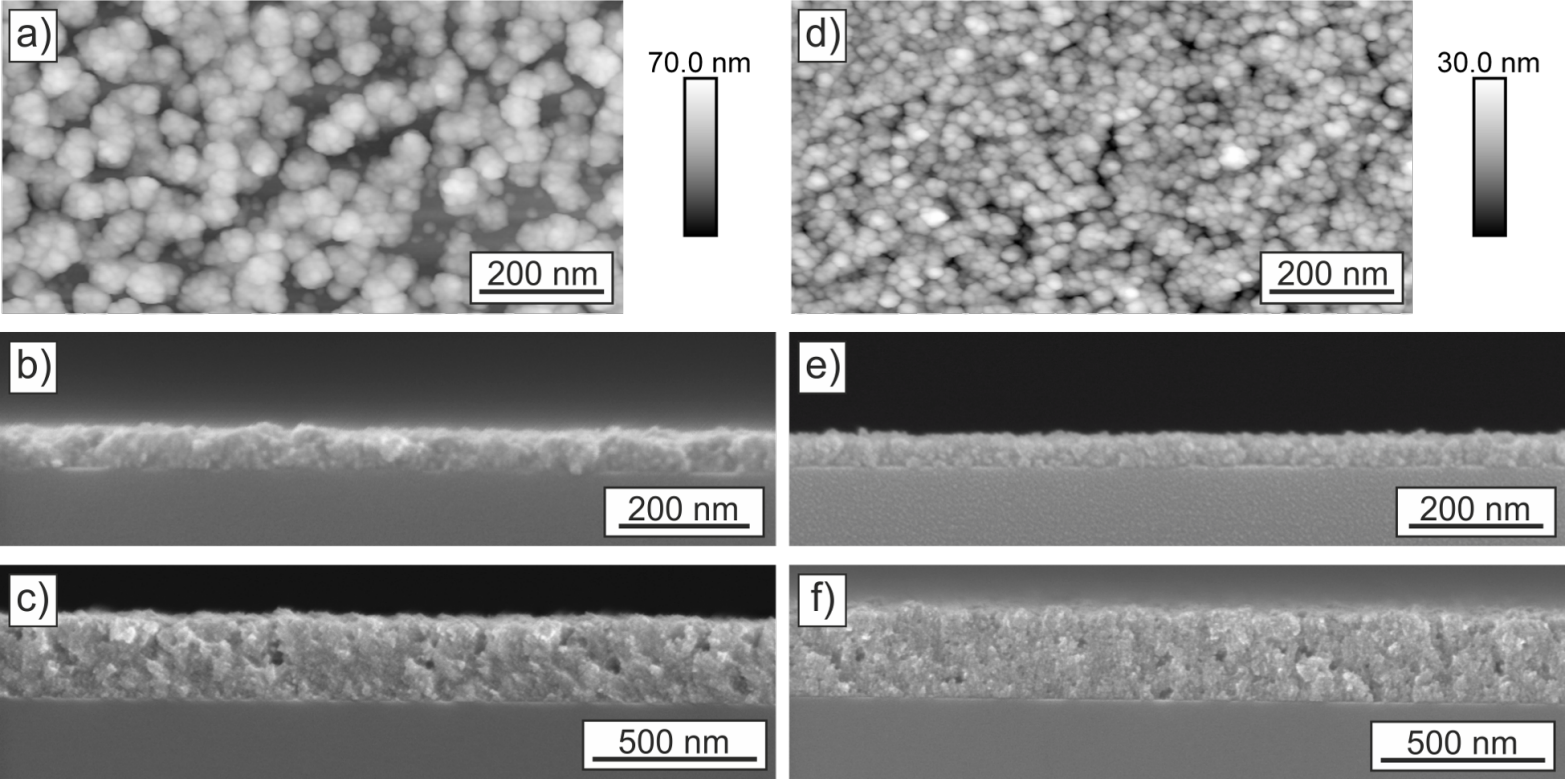
AFM topography images of the ZnO films deposited onto COOH-terminated SAM (a) and PEL (d) after 3 deposition cycles. SEM cross sections of the films after 5 (b,e) and 20 (c,f) deposition cycles on COOH-terminated SAM and PEL (e,f).

Nevertheless, the polarity of the two templates is high enough to promote the deposition of thin ZnO films (Figure 2). The early stage of the deposition after 3 deposition cycles was investigated by AFM measurements (Figure 2a and Figure 2d). The film on the COOH-SAM (Figure 2a) is formed by aggregates with around 50 nm in diameter. The substrate is not completely covered as can be seen from the holes in the ZnO film. Even after five deposition cycles (Figure 2b), these holes are still present. However, after 20 deposition cycles, the ZnO films get more homogeneous and the substrate is covered completely (Figure 2c). Investigation of the deposition of ZnO films on the bare substrate showed that without template only island growth occurs [25]. In comparison, the ZnO films on the highly negatively charged PEL (Figure 2d–f) are more dense and the substrate is covered more homogeneously. This difference can be explained by the different functionalities of the templates. The negative surface charge of the sulfonate-functional groups of the PEL (−115 mV [40]) is high enough to homogeneously attract the dipolar ZnO particles from solution. The result is a closed film even at low numbers of deposition cycles. In contrast, the negative charge density of the carboxylate-terminated SAM is lower (−75 mV [40]). The interaction with the ZnO particles is decreased and less homogeneous films are formed. This is also supported by findings of Wegner et al. [44]. They showed that polymers with sulfonate groups have a high binding tendency to ZnO crystals and inhibit their growth, whereas polymers with carboxylate groups interact less with the crystals. However, the influence of the templates decreases with increasing film thickness. After the template is covered completely with ZnO, the growth rate is only determined by the interaction of the ZnO particles in solution with the ZnO already deposited as film. Therefore, the growth rate is identical on both templates (11 nm per deposition cycle). This allows us to precisely control the film thickness. That films after 3 deposition cycles are slightly thicker than expected, we attribute to a non-uniform ZnO deposition (formation of islands) during first deposition cycles. Our Investigations of films with different thickness showed that the piezoelectric coefficient decreases slightly for film thicknesses above 400 nm (data not shown), which can be attributed to the increasing inhomogeneity of the applied electric fields. Thus, in order to obtain reliable data, we chose the intermediate thickness range on the order of 300 nm. For RF-magnetron-sputtered ZnO films it was reported, that thicker films in the range of 1 µm show an increasing piezo-activity [45]. This trend may arise from a lower density of grain boundaries and a larger crystal size, both of which are achieved by increasing the film thickness. Using our bioinspired growth, the density of the grain boundaries changes only very little with film thickness and therefore it is not necessary to increase the film thickness much above 250 nm in order to achieve highly piezoresponsive layer. For the following investigations, films with similar thicknesses of (265 ± 7) nm on COOH-SAM and (256 ± 9) nm on PEL were used.

**Figure 3:**
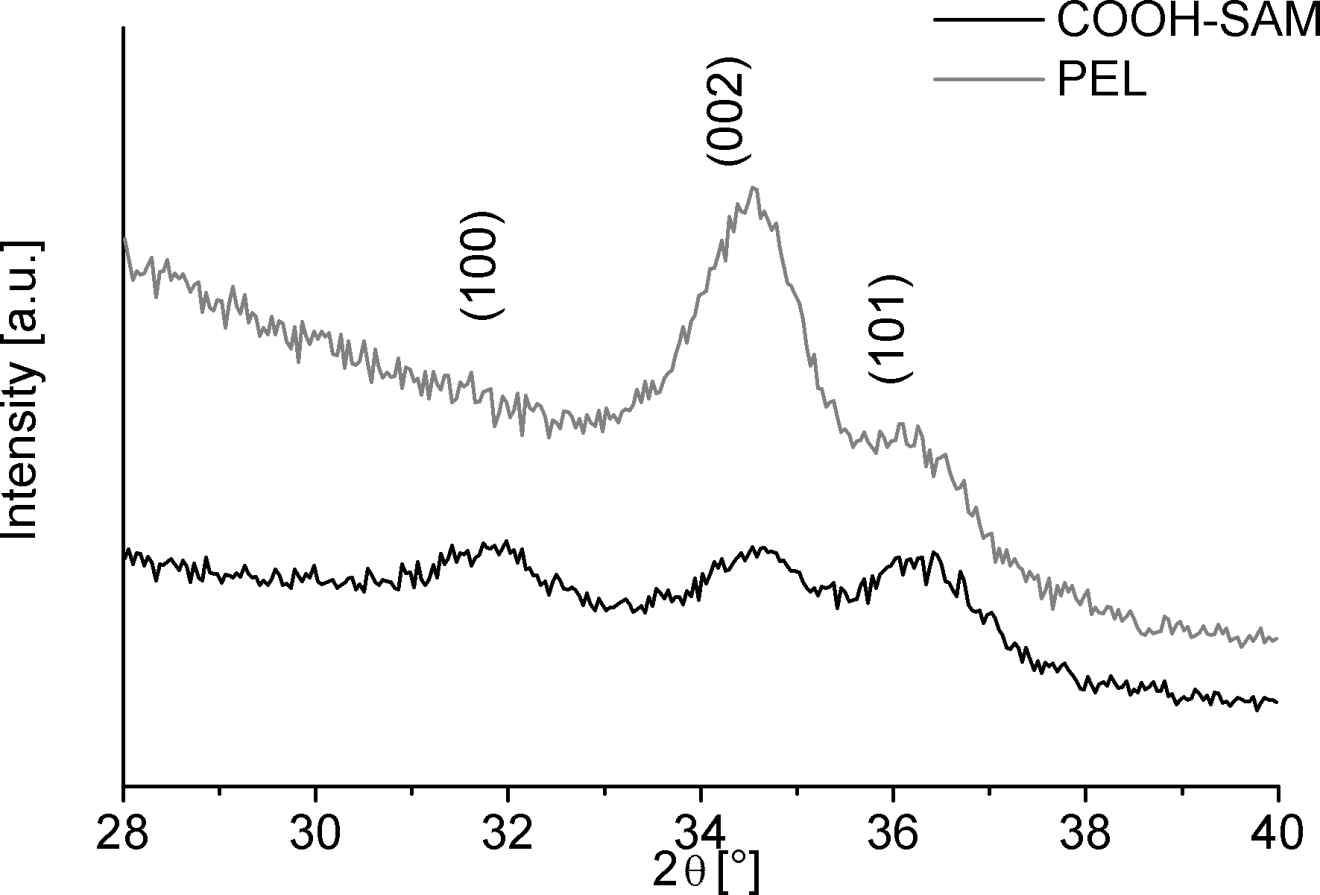
X-ray diffractograms of ZnO films deposited on carboxylate-SAM (black) and PEL (grey). The enhanced relative intensity of the (002)-peak of the PEL sample shows the preferred orientation of the crystallites.

Further influence of the templates on ZnO deposition can be found in the XRD results. The reflections in the diffractograms (Figure 3) at 31.7°, 34.4° and 36.3° 2θ represent the (100), (002) and (101) planes of crystalline ZnO within the hexagonal wurtzite-type structure (JCPDS no. 01-079-0206). The ZnO film deposited on the carboxylate-SAM does not show any preferred orientation (Figure 3a). The film on the PEL on the other hand is textured along the crystallographic (002) direction (Figure 3b). In analogy to the results of Shyue et al., the sulfonate groups should be more negatively charged compared to the carboxylate groups [40]. This can explain the observed texture formation on the PEL. The electrostatic interaction between the ZnO crystallites with the sulfonate groups leads to oriented attachment which is also maintained for higher film thicknesses. The lower charge of the COOH on the other hand is not high enough to prevent the attachment of differently oriented crystallites.

The degree of crystallite orientation influences the piezoelectric activity of the mineralized thin films. To investigate this correlation, PFM measurements were performed on both sample types. The measured electromechanical response has two contributions [46]. The response Δ*z*_ω_ obtained at the frequency ω of the applied voltage is proportional to the effective piezoelectric and electrostrictive constants *d*_eff_ and *M*_333_ via

[1]
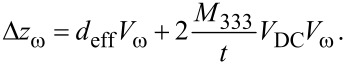


where *V*_ω_ and *V*_DC_ are the driving amplitude and offset of the applied voltage and *t* is the film thickness. To estimate the influence of the electrostriction in the performed measurements, we take the second harmonic of the response into account, which solely depends on the electrostriction via:

[2]
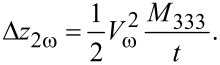


In the setup used in this study, the tip acts as a top electrode. Due to the small tip radius, the electric field is highly inhomogeneous. The interaction between the sample and the tip was described by Kalinin and Bonnell [47]. They found a correlation between the measured piezoelectric coefficient (*d*_eff_) and *d*_33_ to be approximately

[3]
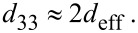


**Figure 4:**
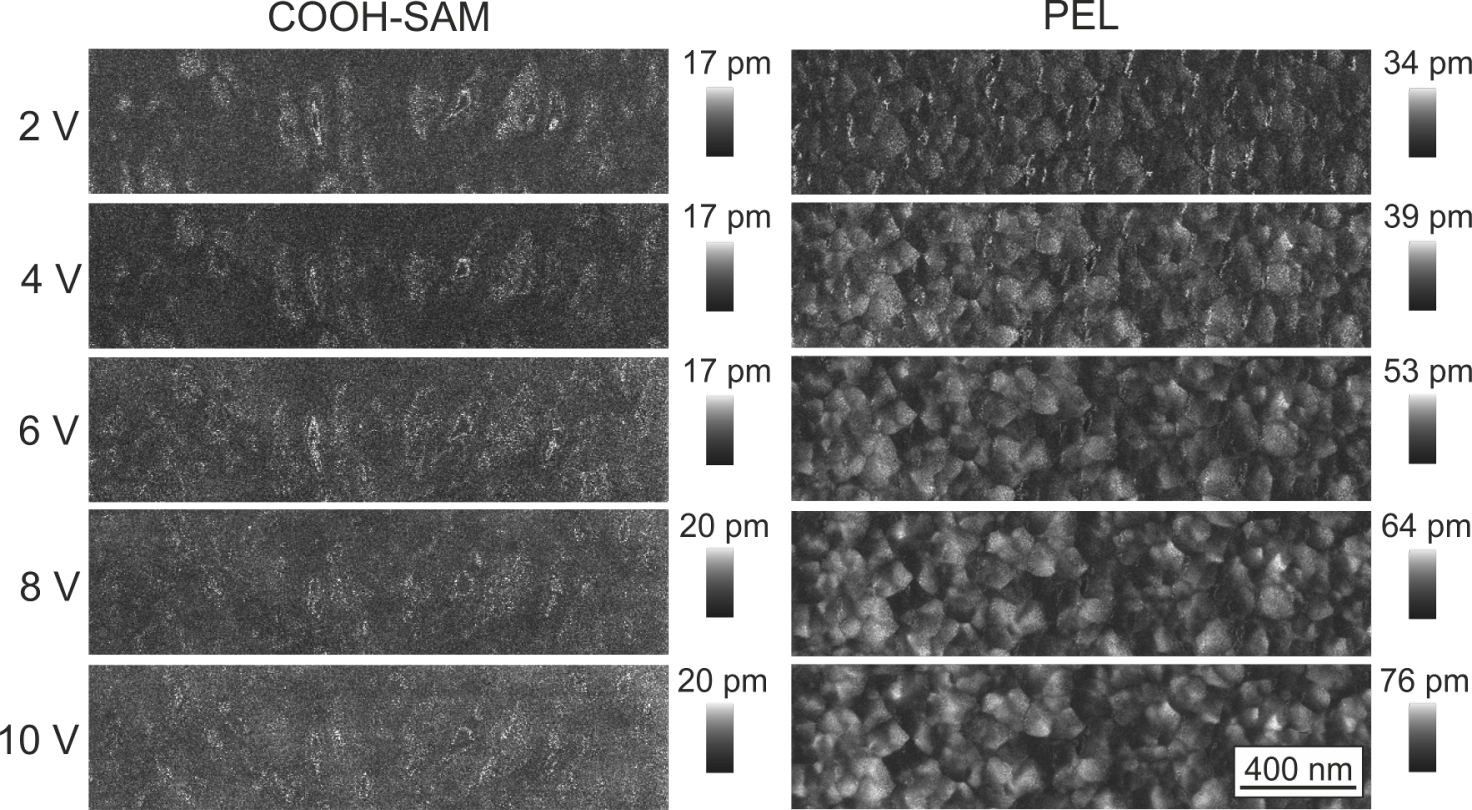
PFM amplitude 1 images obtained by applying voltages of 2 to 10 V of the deposited films on COOH-SAM (left) and PEL (right). The scan area for all images is 2 µm × 0.5 µm.

**Figure 5:**
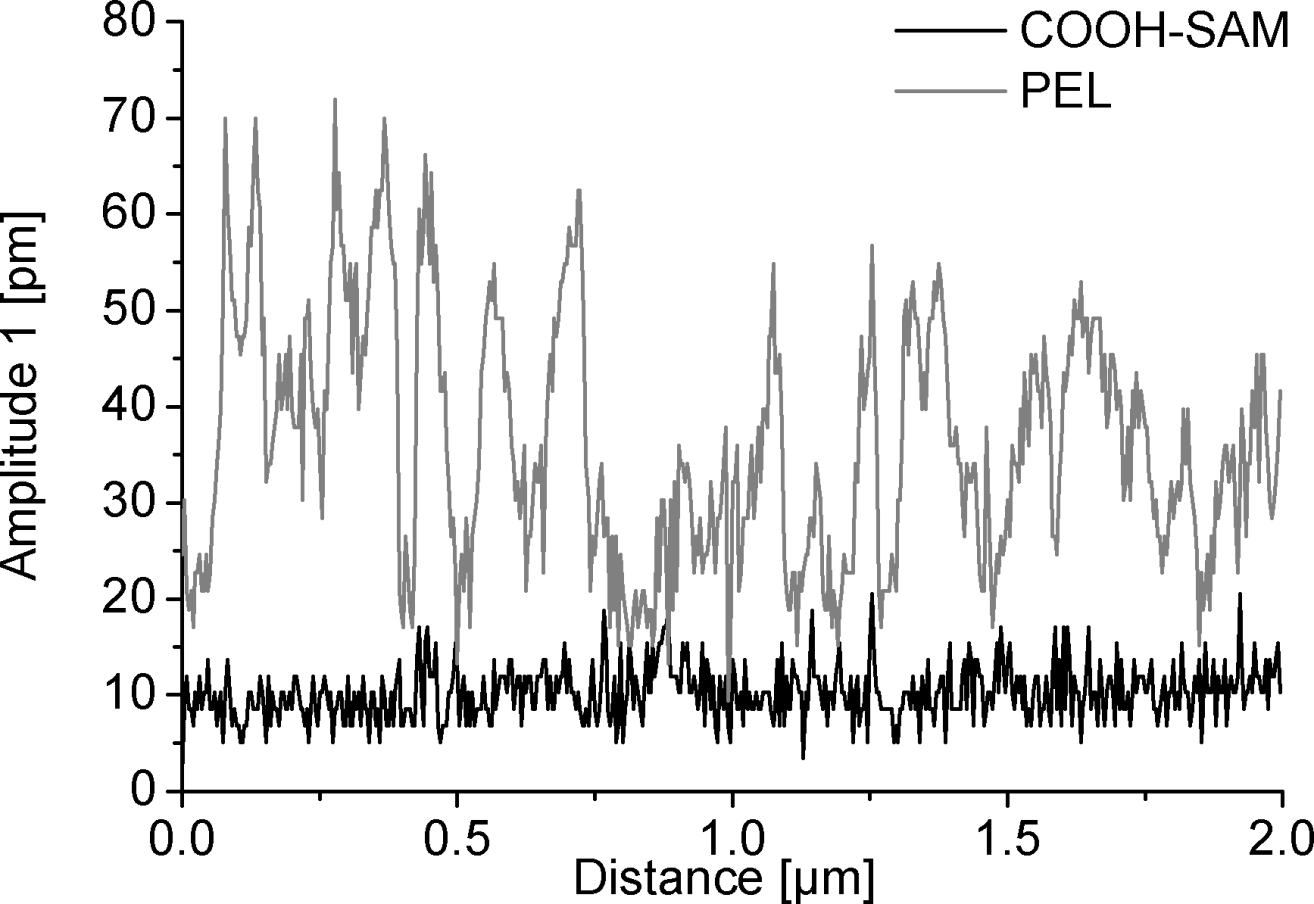
Cross-sections of the amplitude 1 images taken at 10 V for ZnO films deposited on COOH-SAM (black) and PEL (grey).

A first qualitative analysis of the samples can be done by comparing the amplitude 1 images at different drive amplitudes (Figure 4, left side). The brighter the color, the higher is the piezoelectric response of the sample. ZnO on carboxylate-SAMs on the one hand show homogeneous amplitude images with only low contrast. The cross sections (Figure 5, black) show that the average response is in the range of roughly 10 pm. This indicates that the crystallites have different crystallographic orientations so that the overall response is leveled out. Even with increasing drive amplitude, the magnitude of the response stays nearly the same, confirming that no macroscopic piezoelectric response can be measured.

The ZnO on the sulfonate templates on the other hand behaves differently. The crystallites form domains with common orientation. This leads to areas with the same magnitude of deformation, which can also be seen from the cross section in Figure 5 (grey). Additionally, the values for the response are higher compared to the ones obtained on COOH. Here, the mean amplitude is around 30 pm whereas the maximum values for single domains reach up to 70 pm. This correlates well with the XRD results and the (002) texture. The existence of different domains indicates that not all of crystallites are oriented in the same direction. The darker color of some domains suggests that the *c*-axis of these crystallites is tilted with reference to the surface normal. The contrast between the domains increases with increasing drive amplitude. This can be explained by an increase in the magnitude of the piezoelectric response as is expected from Equation 1.

**Figure 6:**
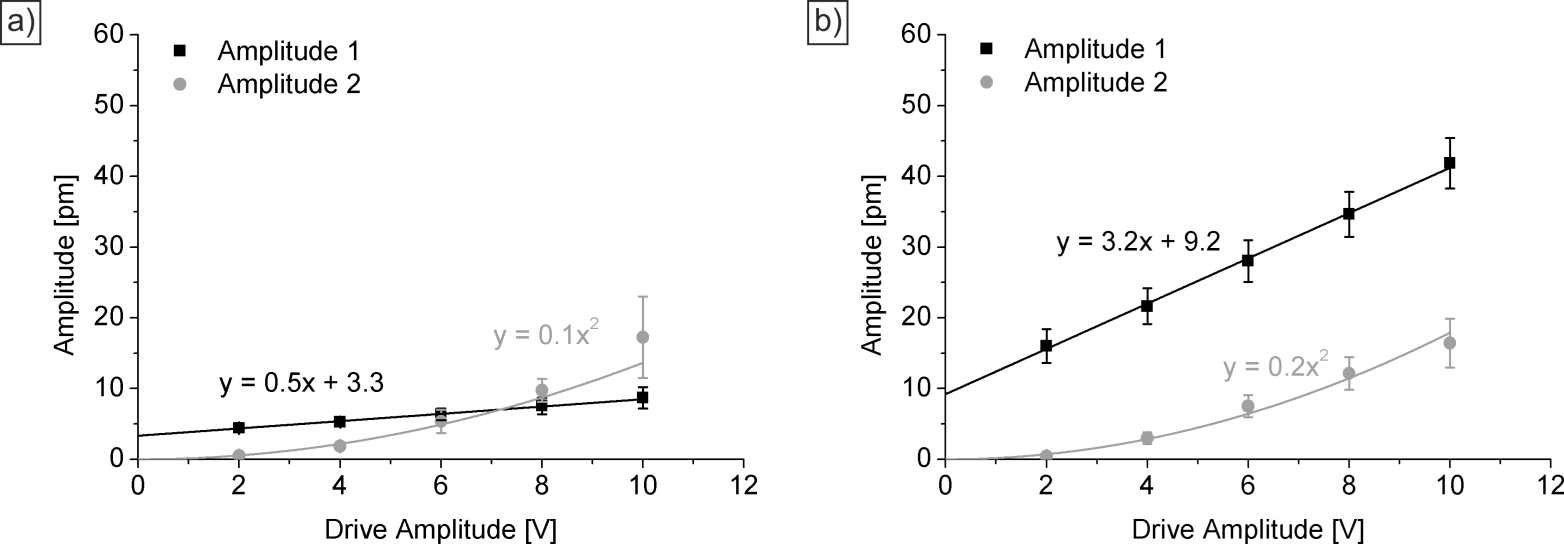
Quantitative analysis of the piezoelectric responses of ZnO films deposited on carboxylate-SAM (a) and PEL (b).

Quantitative analysis is done by plotting the averaged values of the obtained amplitude images against the driving amplitude (Figure 6). For both samples, a low offset is measured at 0 V. This signal corresponds to a background signal that can also be measured on a pure Si wafer [39]. For the sample on the carboxylate-SAM, a linear increase of the amplitude 1 signal can be observed with increasing drive amplitude (compare Equation 1). Also the values for the amplitude 2 signal follow the correlation from Equation 2. On the other hand, it can be seen that the amplitude 2 signal is higher compared to the amplitude 1 signal at higher voltages. This indicates that the electrostriction dominates the electromechanical response of the sample. These results confirm the XRD results, where the ZnO films do not exhibit a (002) texture. Consequently, the piezoelectric coefficient calculated is quite small (*d*_eff_ = 0.5 pm V^−1^) and in the range obtained for the pure silicon substrate [39].

For the ZnO films on sulfonate-terminated substrates, again, a behavior that is consistent with Equations 1 and 2 is found. In contrast to the sample on COOH, the amplitude 1 signal is higher compared to the amplitude 2 signal at all voltages. This indicates the pronounced piezoelectric activity of the films due to their (002) texture. This also reflects in the value obtained for *d*_eff_ of 3.2 pm V^−1^ (*d*_33_ = 6.4 pm V^−1^). This value is in the range or higher than the one of other oriented, ZnO thin films prepared by RF magnetron sputtering (2–13 pm V^−1^) [14] or sol–gel process (5 pm V^−1^) [15]. Since XRD data show that the crystal orientation is not perfect, the obtained *d*_33_ is slightly lower compared to the values obtained on single crystalline ZnO of 9.9 pm V^−1^ [48–49].

## Conclusion

These results show that we are able to tune the piezoelectric performance of ZnO thin films by applying processes inspired by nature. By choosing differently functionalized organic templates, it is possible to influence the deposition behavior of ZnO from solution at 60 °C. The morphology of the films at low numbers of deposition cycles is more homogeneous the higher the negative surface charge of the template. However, this influence is reduced for higher numbers of deposition cycles. Additionally, the surface charges determines whether the film is textured (PEL) or non-textured (COOH-SAM). By choosing PEL surfaces instead of COOH-surfaces the piezoelectric coefficient (*d*_33_) can be increased from lower than 1.0 pm V^−1^ to 6.4 pm V^−1^. This leads to responses comparable to ZnO structures till now only obtained by RF magnetron sputtering [14] or after high temperature treatment (e.g., at 500 °C) [15].

## Experimental

### Template preparation

Boron-doped Si (100) wafers were cleaned first in Milli-Q water and then in acetone/ethanol 1:1 for 10 min in an ultrasonic bath. Afterwards, they were treated for 10 min in an O_2_-plasma with 30 W, followed by another cleaning in Milli-Q water in ultrasound (10 min). In-between the different steps, the wafers were dried with N_2_.

**Carboxylate-SAMs** were prepared according to Hoffmann et al. [50]. After the cleaning procedure, a 3-aminopropyltriethoxysilane-SAM (APTES, Acros Organics, 99%) was prepared and functionalized with a 143 mm solution of succinic anhydride (Sigma-Aldrich, ≥99%) in 1-methyl-2-pyrrolidone (Sigma-Aldrich, ≥98.0%).

**Polyelectrolyte layers** were deposited according to Lipowsky et al. with a dipping robot DR 3 from Riegler & Kirstein, Germany [26]. Solutions of poly(styrene sulfonate) (PSS, Sigma-Aldrich, *M* ≈ 70,000 g mol^−1^), poly-L-glutamic acid (PLGA, Sigma-Aldrich, *M* = 15,000–50,000 g mol^−1^) and poly-L-lysine hydrobromide (PLL, Sigma-Aldrich, *M* = 15,000–30,000 g mol^−1^) in Milli-Q water with a concentration of 1 mg mL^−1^ were prepared. The pH of the PLL solution was adjusted to 9 with 0.3 m KOH. The sequence of the layer-by-layer deposition was (PLL + PLGA)_5_ + PLL + PSS. The substrates were dipped into the polyelectrolyte solutions for 20 min, followed by several washing steps in Milli-Q water.

### ZnO mineralization

For all solutions, methanol (BASF, VLSI selectipur) was used as solvent. Stock solutions with 34.02 mm Zn(CH_3_COO)_2_∙2H_2_O (Sigma-Aldrich, ≥99.0%), 25.71 mm poly(vinylpyrrolidone) (PVP, Sigma-Aldrich, *M*_w_ ≈ 10,000 g mol^−1^, batch BCBJ4889V) and 75 mm tetraethylammonium hydroxide (TEAOH, Sigma-Aldrich, 1.5 m in methanol) were prepared. The PVP and zinc acetate solutions were mixed and the TEAOH was added drop-wise with a peristaltic pump under gentle stirring. The final composition was [Zn^2+^] = 11.34 mm, [PVP] = 8.57 mm and [TEAOH] = 25 mm.

The coated wafers were each immersed in 1 mL of this solution. The reaction took place at 60 °C in an oil bath for 90 min. Afterwards the substrates were thoroughly washed in methanol. The procedure in this paragraph was repeated several times to increase the film thickness.

### Characterization

For **scanning electron microscopy (SEM) measurements** a DSM 982 GEMINI field-emission SEM with a thermal Schottky-field emitter at a working distance of 2 mm and an acceleration voltage of 3 kV was used. Cross-sections were prepared by sputtering with 80:20 Pt/Pd.

**X-ray diffractometry (XRD)** measurements were performed on a PANalytical X'Pert MPD with Cu Kα radiation (45 kV; 40 mA) in parallel beam geometry. The diffractograms were recorded in a range of 28–40° with a step size of 0.04°. The samples were tilted by an angle of ψ = 5° to prevent reflections from the substrate.

**PFM measurements** were carried out on a Bruker Multimode 8 with a Nanoscope 5 controller in contact mode. Commercially available MESP-RC tips from Bruker were used as top electrodes. The ZnO samples were glued to metallic sample holders with a graphite tape and contacted with silver paste. Calibration of the photodiode was performed by measuring the force distance curves and calculating the deflection sensitivity.

Height images were flattened 1st order with the software Nanoscope Analysis v. 1.50 (Bruker).

In order to measure the piezoresponse of the samples, an AC voltage with a frequency of 20 kHz was applied between the tip and the sample (tip grounded). By applying an alternating current, the sample starts to oscillate and the signal can be analyzed via a lock-in amplifier. The driving amplitude was varied between 2 and 10 V. Amplitude 1 and 2 signals were recorded simultaneously corresponding to the first and second harmonic response, respectively. The obtained signal was averaged over the complete image and the values were plotted against the driving amplitude. From Equation 1 follows that the slope of the resulting Amplitude 1 curve gives the piezoelectric coefficient *d*_eff_. By applying Equation 3, *d*_33_ can be calculated.
